# Development of a population pharmacokinetics and pharmacodynamics model of glucarpidase rescue treatment after high-dose methotrexate therapy

**DOI:** 10.3389/fonc.2023.1003633

**Published:** 2023-01-30

**Authors:** Yutaka Fukaya, Toshimi Kimura, Yukihiro Hamada, Kenichi Yoshimura, Hiroaki Hiraga, Yuki Yuza, Atsushi Ogawa, Junichi Hara, Katsuyoshi Koh, Atsushi Kikuta, Yuhki Koga, Hiroshi Kawamoto

**Affiliations:** ^1^Department of Pharmacy, Tokyo Women’s Medical University Hospital, Tokyo, Japan; ^2^Department of Pharmacy, Juntendo University Hospital, Tokyo, Japan; ^3^Center for Integrated Medical Research, Hiroshima University Hospital, Hiroshima, Japan; ^4^Department of Musculoskeletal Oncology, National Hospital Organization Hokkaido Cancer Center, Sapporo, Japan; ^5^Department of Hematology and Oncology, Tokyo Metropolitan Children’s Medical Center, Tokyo, Japan; ^6^Department of Pediatrics, Niigata Cancer Center Hospital, Niigata, Japan; ^7^Department of Pediatric Hematology/Oncology, Osaka City General Hospital, Osaka, Japan; ^8^Department of Hematology/Oncology, Saitama Children’s Medical Center, Saitama, Japan; ^9^Department of Pediatric Oncology, Fukushima Medical College, Fukushima, Japan; ^10^Department of Perinatal and Pediatric Medicine, Kyushu University, Fukuoka, Japan; ^11^Department of Pediatric Oncology, National Cancer Center Hospital, Tokyo, Japan

**Keywords:** glucarpidase, methotrexate - adverse effects, safety, pharmacokinetics, pharmacodynamics

## Abstract

**Introduction:**

Glucarpidase (CPG2) reduces the lethal toxicity of methotrexate (MTX) by rapid degradation.

**Methods:**

In this study, a CPG2 population pharmacokinetics (popPK) analysis in healthy volunteers (phase 1 study) and a popPK-pharmacodynamics (popPK-PD) analysis in patients (phase 2 study, *n* = 15) who received 50 U/kg of CPG2 rescue for delayed MTX excretion were conducted. In the phase 2 study, the first CPG2 treatment at a dose of 50 U/kg was intravenously administered for 5 min within 12 h after the first confirmation of delayed MTX excretion. The second dose of CPG2, with a plasma MTX concentration >1 μmol/L, was administered to the patient more than 46 h after the start of CPG2 administration.

**Results:**

The population mean PK parameters (95% CI) of MTX, obtained from the final model *post hoc*, were estimated as follows: *CLr_MTX_* = 2.424 L/h (95% CI: 1.755–3.093), *Vc_MTX_* = 12.6 L (95% CI: 10.8–14.3), *Vp_MTX_* = 2.15 L (95% CI: 1.60–2.70), and *α* = 8.131 x 10^5^ (4.864 x 10^5^-11.398 x 10^5^). The final model, including covariates, was *CLr_MTX_* (L/h): 3.248 x *Body Weight*/*Serum creatinine/*60 (CV 33.5%), *Vc_MTX_* (L): 0.386 x *Body Weight/body surface area* (CV 29.1%), *Vp_MTX_* (L):3.052 x *Body Weight*/60 (CV 90.6%), and *α* (L/h): 6.545 x 10^5^ (CV 79.8%).

**Discussion:**

These results suggest that the pre-CPG2 dose and 24 h after CPG2 dosing were the most important sampling points in the Bayesian estimation of plasma MTX concentration prediction at 48 h. These CPG2-MTX popPK analysis and Bayesian estimation of rebound in plasma MTX concentrations are clinically important to estimate >1.0 μmol/L 48 h after the first CPG2 dosing.

**Clinical trial registration:**

https://dbcentre3.jmacct.med.or.jp/JMACTR/App/JMACTRS06/JMACTRS06.aspx?seqno=2363, identifier JMA-IIA00078 and https://dbcentre3.jmacct.med.or.jp/JMACTR/App/JMACTRS06/JMACTRS06.aspx?seqno=2782, identifier JMA-IIA00097.

## 1 Introduction

Methotrexate (MTX) is an antineoplastic drug that inhibits fatty acid metabolism. Combination chemotherapy with high-dose MTX (HD-MTX) has been established as the standard regimen for acute lymphoblastic leukemia, malignant lymphoma, and osteosarcoma. HD-MTX causes adverse effects such as nephrotoxicity, hepatotoxicity, severe mucositis, and pancytopenia at a frequency of 1%–10% (1, 2). Treatments such as plasmapheresis, dialysis, and high-dose leucovorin can be used to prevent fatal MTX toxicity; however, these treatments have limited efficacy (2). Nephrotoxicity caused by HD-MTX seems to result mainly from the inhibition of renal tubular excretion of MTX.

In a physiological pathway, most MTX is excreted through the urine as an intact drug by the organic anion transporter-3 (3–5), and less than 10% of MTX is metabolized to relatively inactive 7-hydroxymethotrexate. Other metabolites (less than 5% of MTX) include an inactive metabolite of 2,4-diamino-N10-methylpteroic acid (DAMPA) (3, 4, 6, 7). Glucarpidase (CPG2) is an enzymatic drug (a 390-amino acid homodimer protein with a molecular weight of 83 kDa isolated from the *Pseudomonas* species) that directly decomposes MTX into DAMPA and glutamate (8). Administration of CPG2 is an effective option as an alternative pathway of MTX metabolism if MTX induced acute renal failure and decreased the renal clearance of MTX (8). In Europe and the United States, CPG2 is the only standard drug approved as a therapeutic agent for reducing MTX toxicity caused by delayed MTX excretion. CPG2 rapidly degrades MTX and reduces plasma MTX concentrations by >95% within 15 min (9–13). A 50-U/kg dose of CPG2 is used in clinical treatment as a theoretically sufficient dose to reduce high plasma MTX concentrations. In an open-label and single-site study, Phillips et al. reported a safe and effective dose of 50 U/kg of CPG2 (14). Several reports have suggested efficacy at doses between 15 and 70 U/kg of CPG2; however, only a few dose-finding studies of CPG2 have been conducted in humans.

We conducted a phase 1 study in which two doses of CPG2 (50 and 20 U/kg) were administered to healthy volunteers (15). Safety was confirmed when 50 U/kg of CPG2 was administered at 48-h intervals. As it is possible that the degradation effect of MTX may be diminished by low doses of CPG2, we set the recommended dose of CPG2 to 50 U/kg and conducted a phase 2 study in Japanese patients receiving HD-MTX.

In this study, we aimed to develop a CPG2 population pharmacokinetics (popPK) analysis in healthy volunteers and a popPK and pharmacodynamics (PD) analysis (popPK-PD) in patients who received CPG2 rescue for delayed MTX excretion. The rebound in plasma MTX concentrations after the administration of CPG2 has been reported (8). Based on the concept of model-informed precision dosing (MIPD), the popPK-PD final model was evaluated using a Bayesian estimation to predict the plasma MTX concentrations.

## 2 Materials and methods

### 2.1 Subjects

#### 2.1.1 Phase 1 study

A phase 1 open-label, randomized two-dose (20 and 50 U/kg) study was conducted in healthy volunteers, and a parallel PK study of CPG2 was launched at Hamamatsu University School of Medicine. This study was conducted between November 2011 and January 2012.

#### 2.1.2 Phase 2 study

A phase 2 multicenter collaborative, single-arm uncontrolled study was conducted in patients with delayed excretion of MTX after HD-MTX treatment for cancer. This study was conducted between December 2012 and February 2016 at eight Japanese medical facilities. After treatment with HD-MTX (≥1 g/m^2^), CPG2 (50 U/kg) was administered to patients with abnormally high plasma MTX concentrations far above the suggested critical level or elevated serum creatinine levels.

The selection criteria for the treatment of CPG2 were defined by plasma MTX concentrations and time after administration, depending on the status of the patients (**Table 1**).

**Table 1:** Selection criteria for the treatment of CPG2.

Plasma MTX concentrations and time after MTX administration
≥50 μmol/L (22 h)
≥5 μmol/L (40 h)
≥2 μmol/L (46 h)
≥1 μmol/L (40 h and patients with acute renal failure^a^)
≥0.4 μmol/L (46 h and patients with acute renal failure^a^)
≥0.3 μmol/L (70 h and >3.5 g/m^2^ of MTX dose)
≥0.1 μmol/L (70 h and 1–3.5 g/m^2^ of MTX dose)

CPG2, glucarpidase; MTX, methotrexate.

a Acute renal failure is defined as meeting either of the following (1) and (2): (1) after 12 h of MTX administration, the serum creatinine level was above the upper limit of the reference, or creatinine clearance or glomerular filtration rate (either calculated or measured) was <70 ml/min; (2) increase the serum creatinine level by two times or more before MTX administration, or by a factor of 1.5, for two consecutive times and increasing.

Phase 1 and 2 studies were performed in accordance with the Declaration of Helsinki and Guidelines for Good Clinical Practice, with approval from the Human Institutional Review Boards or independent ethics committees for each trial. This investigator-initiated clinical trial was supported by the Center for Clinical Trials of Japan Medical Association (JMACCT). Phase 1 and 2 studies were registered with the JMACCT Clinical Trial Registry (identifiers: JMA-IIA00078 and JMA-IIA00097).

### 2.2 Dosing regimen, blood sampling, and determination of plasma concentrations of CPG2 and MTX

**Figure 1:**
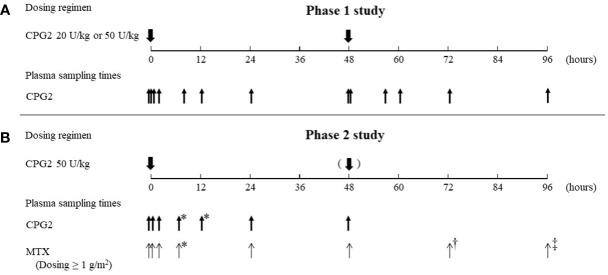
Summary of the dosing regimen of CPG2 and blood sampling of CPG2 and MTX. CPG2, glucarpidase; MTX, methotrexate. **(A)** Blood sampling of CPG2 in the phase 1 study. First dosing of CPG2: before dosing, at 5 and 15 min, and 2, 8, 12, 24, and 48 h (before second dosing of CPG2) after dosing. Second dosing of CPG2: 5 min and 8, 12, 24, and 48 h after dosing. **(B)** Blood sampling of CPG2 in the phase 2 study. First dosing of CPG2: before dosing, at 20 min, and 2, 5–8^*^, 12–20^*^, 24, and 48 h after dosing. Blood sampling of MTX. First dosing of CPG2: before dosing, at 20 min, and 2, 5–8^*^, 24, 48, 72†, and 96‡ h after dosing. *Before leucovorin dosing; †24 h after and dosing of CPG2; ‡ 48 h after second dosing of CPG2.

The sampling times and dosing regimens of CPG2 and MTX for the phase 1 and 2 studies are summarized in **Figure 1**.

#### 2.2.1 Phase 1 study

A dose of 50 or 20 U/kg of CPG2 was administered intravenously over 5 min. Eight healthy volunteers were allocated to the two-dose groups. All participants received the same second dose of CPG2, 48 h after the first dose.

#### 2.2.2 Phase 2 study

The first CPG2 treatment at a dose of 50 U/kg was intravenously administered for 5 min within 12 h after the first confirmation of delayed MTX excretion. The second dose of CPG2 was administered to patients with a plasma MTX concentration >1 μmol/L more than 46 h after the start of CPG2 administration. Supportive care such as leucovorin rescue was continued until a plasma MTX concentration <0.1 μmol/L was obtained; however, CPG2 was not administered for a third time.

### 2.3 Assay methods

All blood samples were centrifuged for 15 min to separate the plasma. The plasma MTX concentrations for clinical decision-making regarding CPG2 therapy were immediately analyzed at each hospital using a routine assay method. Measurement of the plasma MTX concentrations at each hospital is a commercial immunoassay, and there is a possibility of cross-reactivity between MTX and DAMPA (16). The same samples were also analyzed at the central laboratory using a quantitative validated analytical method by high-performance liquid chromatography (HPLC) that permits the separation of MTX metabolites such as DAMPA. Only MTX concentrations measured by HPLC were used for the PK analysis. Plasma samples of MTX and CPG2 were stored at −70°C and −80°C, respectively, until central laboratory analysis. These samples were analyzed by Shin Nippon Biomedical Laboratories, Ltd. (Wakayama, Japan).

The HPLC analysis of plasma MTX concentration was performed using the mobile phase that consisted of formic acid (0.1% vol) and methanol (90:10). The analytical column was an octadecyl silyl (ODS) column (Hydrosphere C18 S-5, 2.0 × 75 mm I.D., YMC CO., Kyoto, Japan). The column temperature was set at 40°C. The flow rate of the mobile phase was set to 0.5 ml/min. MTX-d3 (Toronto Research Chemicals, Inc. Toronto, Canada) was used as an internal standard. The lower limit of quantification (LLOQ) and the upper limit of quantification (ULOQ) of plasma MTX concentrations were 0.5 and 400 ng/ml, respectively.

Plasma CPG2 concentrations were determined using enzyme-linked immunosorbent assay (ELISA) (15). All analytical methods were validated according to the Guidelines on Bioanalytical Method Validation in Pharmaceutical Development in Japan. The calibration ranges for CPG2 were defined by the LLOQ and ULOQ with seven calibration standards of different concentration levels, including the LLOQ and ULOQ, with a correlation coefficient of ≥0.990. The LLOQ and ULOQ of plasma CPG2 concentrations were 1 ng/ml and 640 μg/ml, respectively.

### 2.4 Development of the popPK and popPK-PD models

First, the popPK model of CPG2 was determined from the phase 1 and 2 studies. We then constructed a popPK-PD model that incorporated the degradation reaction of CPG2 into the metabolic clearance of MTX using the Michaelis–Menten equation in the phase 2 study. Simultaneous analysis of all concentration–time and patient physiological data was performed using Phoenix 64 NLME 7.0 (Pharsight Corp., Mountain View, CA, USA), a computer program developed for popPK and popPK-PD analyses. PopPK parameters and their variability were estimated using first-order conditional estimation extended least-squares (FOCE-ELS) methods.

### 2.5 Development of the CPG2 popPK model

In the first step, popPK analysis of CPG2 was developed in the phase 1 and 2 studies. One- and two-compartment intravenous infusion models with first-order elimination of CPG2 were used to describe the plasma concentration–time courses. Additive and proportional (exponential) error models were compared for interindividual variability in all PK parameters and residual variability in drug concentrations. The additive error model was used for residual variability, which is expressed as follows:


Cij=Cij*+ϵij

where *Cij* is the *i*th measured plasma concentration in the *j*th individual, Cij* is the estimated plasma concentration (as observed for PK data), and *ε;ij* is the independent identically distributed statistical error with a mean of zero and a variance of *σ;*^2^. Exponential interindividual variability models were invoked for all PK parameters as follows:


$$\theta_{i}=tv\theta \times exp\left(n_{i,\theta }\right)$$

where *θ*_1_ is the individualized estimated parameter, *tvθ* is the typical value of the parameter in the population, and *n_i_*,*_θ_* is a random variable with a mean of zero and a variance of *ω*^2^. In the fitting process, subject demographic and biochemical data were used as covariables in the population model: body weight, height, body surface area (BSA), and age (only in the phase 2 study). The candidate covariate was screened through the simultaneous incorporation of an allometric function on all typical values of PK parameters, such as clearance (*tvCL_CPG2_*) and distribution volume (*tvV_CPG2_*). These were expressed as follows:


$$CL_{CPG2}=tvCL_{CPG2}\times covariate^{\theta 1}$$

$$V_{CPG2}=tvV_{CPG2}\times covariate^{\theta 2}$$

where *θ*_1_ and *θ*_2_ are the intercept and slope parameters, respectively. An objective function value (OFV; negative value of twice the log-likelihood difference: −2 L.L.d) was calculated for each model in this regression. Covariates such as height, body weight, BSA, age, serum creatinine, and creatinine clearance in each model were determined using the likelihood ratio test based on the degree of freedom of parameters and change in the OFV. Covariates were included in the full model when the likelihood ratio test was decreased in OFV greater than 10.828 (*p*-value < 0.001). Covariates without multicollinearity were basically considered in the full model. When there was no change in degrees of freedom and multiple covariates were selected, the full model was selected with reference to Akaike’s information criterion. The definition of successful in each model analysis was that the standard deviation of all parameters was calculated and the 95% confidence intervals of the parameters were positive values. Each covariate was added using a forward–backward stepwise in the popPK model to validate the final model. The final model determined the most significant model in the likelihood ratio test.

### 2.6 Development of the popPK-PD model for MTX decomposition by CPG2

**Figure 2:**
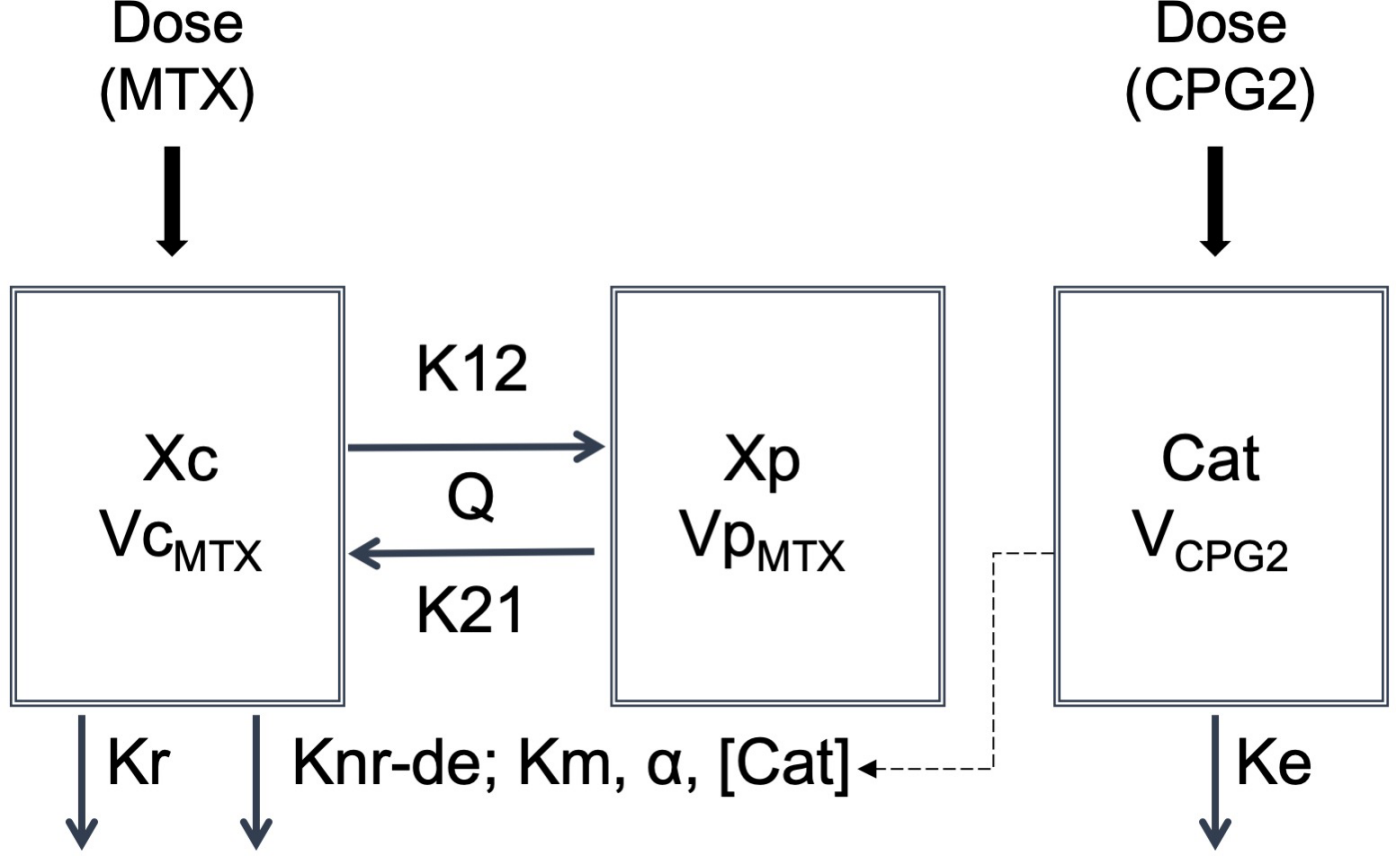
Structure of the popPK-PD model of decomposing MTX by CPG2. Cat, catalyst; *Ke*, elimination rate constant of glucarpidase; *Knr-de*, degradation rate constant; *Km*, Michaelis–Menten constant; *Kr*, renal elimination rate constant of methotrexate; *K*12, distribution rate constant for transfer from central to peripheral; *K*21, distribution rate constant for transfer from peripheral to central; *Q*, intercompartment clearance of *Vc_MTX_* and *Vp_MTX_*; *V_CPG2_*, volume of distribution of glucarpidase; *Vc_MTX_*, central compartment volume of distribution of methotrexate; *Vp_MTX_*, peripheral compartment volume of distribution of methotrexate; *Xc*, amount of the central compartment of methotrexate; *Xp*, amount of the peripheral compartment of methotrexate; *α*, conversion constant.


In the second step, CPG2-MTX and popPK-PD analyses were performed based on the known popPK properties of MTX. The default structural model of MTX was a two-compartment model using the following differential equations (**Figure 2**):

(1) $$\begin{array}{l} dXc/dt=-\left(Kr+Knr‐de+K12\right)\times \\ Xc+K21\times Xp \end{array}$$

dXp/dt=K12×Xc−K21×Xp

where *Xc* and *Xp* are the amounts of MTX in the central and peripheral compartments, respectively. *Kr* and *Knr-de* are elimination constants from the central compartment by renal excretion and metabolic pathways, respectively. *K*12 and *K*21 are the intercompartmental constants between the central and peripheral compartments, respectively. In this differential equation, *dXc*/*dt*=−(*Kd* ×*Xc*) represents the decomposition of MTX by CPG2 as a catalyst. CPG2 is an enzyme that hydrolyzes MTX to inactive metabolites and is characterized by the Michaelis–Menten equation. Therefore, the metabolism of MTX is as follows (17):


$$dXc/dt=-V_{max}\times \left[\text{Xc}\right]/\left(Km+\left[Xc\right]\right)$$

where *V_max_* is the maximum rate of decomposition, *Km* (18) is the Michaelis–Menten constant of MTX hydrolysis by CPG2, and [*Xc*] is the plasma MTX concentration. *V_max_* is also a constant for the interaction of the enzyme with its substance and has units of mass/time. Therefore, it can be rearranged based on mass balance as follows:


$$\begin{array}{l} V_{max}\left(mass/time\right)=\left[catalyst\right]\times \;\\ \alpha \left(mass/volume\times volume/time\right) \end{array}$$

where *α* is the conversion constant between *V_max_* and the catalyst concentration for a patient-specific parameter, which is the same as the individual clearance.

Equation (1) was rearranged using a constant *α* and the catalyst concentration, as follows:


$$\begin{array}{l} dXc/dt=-\left(Kr+K12\right)\times \\ Xc+K21\times Xp-\alpha \times \left[catalyst\right]\times \\ \left[Xc\right]/\left(Km+\left[Xc\right]\right) \end{array}$$

The catalyst concentration was the plasma CPG2 concentration, which was calculated using the patient’s individualized *CL_CPG2_* and *V_CPG2_*, obtained *post hoc* in the popPK analysis of the phase 2 study. To build the popPK model, renal clearance was calculated using the micro rate constants of *Kr* and distribution volume in the central compartment (*CLr*_*MTX*_ = *Kr* × *Vc*_*MTX*_ ) to incorporate physiological covariates such as height, body weight, BSA, age, serum creatinine, and creatinine clearance. The intercompartmental clearance, *Q*, of MTX was defined as follows: *Q*=*K*12×*Vc*_*MTX*_=*K*21×*Vp*_*MTX*_ . Model selection and determination of the final model were performed using the same process as in CPG2. A proportional error model was used to determine the residual variability of plasma MTX concentrations: *Cij*=Cij*×(1+*ϵij*) .

### 2.7 Model evaluation

The general goodness-of-fit of the final popPK model of CPG2 and of the CPG2-MTX popPK-PD model was evaluated using the scatter plots of observed plasma concentrations versus predicted plasma concentrations (PRED) based on the mean popPK parameters or individual predicted plasma concentrations (IPRED) using Bayesian estimation and the plots of CWRES versus the relative elapsed time after drug administration. A bootstrap assessment of the CPG2 popPK final model and CPG2-MTX popPK-PD final model was conducted using Phoenix 64 NLME 7.0 (with 1,000 samples). A visual predictive check (VPC) was used to assess the performance of the final popPK model of CPG2. The 5th, 50th, and 95th percentiles of the distribution of the observed and 1,000 simulated plasma concentrations were calculated and graphically compared.

### 2.8 Sampling strategy and prediction performance using Bayesian estimation

The sampling strategy and prediction performance of plasma MTX concentrations 48 h after the first administration of CPG2 were investigated using the CPG2-MTX popPK-PD final model by Bayesian estimation in Phoenix 64 NLME 7.0.

To evaluate the best sampling points for the estimation of plasma MTX concentrations at 48 h, the combined multi-sampling MTX concentrations (before CPG2 dosing plus at 20 min, 2 h, 5–8 h, and 24 h after CPG2 dosing) were used. The predicted performance was evaluated using the mean error (ME), mean absolute error (MAE), and root-mean-squared error (RMSE). The ME, MAE, and RMSE were calculated using the predicted plasma MTX concentrations and observed plasma MTX concentrations. We also verified the hit ratio of the estimated plasma MTX concentrations to the observed plasma MTX concentrations over or under 1.0 μmol/L, which is the borderline of an additional dose of CPG2, 48 h after the first CPG2 dosing.

## 3 Results

### 3.1 Patient characteristics in the phase 1 and 2 studies

Sixteen healthy subjects were randomly allocated to two cohorts (20 U/kg, *n* = 8 or 50 U/kg, *n* = 8) in the phase 1 study. The participant demographics and baseline characteristics did not differ significantly between the two cohorts. The study participants were men (100%) with a mean age of 24.9 years (range, 20–40), height of 169.6 cm (range, 162.6–184.8), and body weight of 60.9 kg (range, 54.3–66.9). The demographic characteristics of the participants in the phase 2 study are shown in **Table 2**.

**Table 2:** Demographic data in phase 2 (*n* = 15).

Characteristics	Number	Percentage (%)
Male	9	60.0
Female	6	40.0
Osteosarcoma	9	60.0
Acute lymphocytic leukemia	3	20.0
Non-Hodgkin’s lymphoma	2	13.3
Medulloblastoma	1	6.7
Value (units)	Median	Range
Age (years)	15	1–75
Height (cm)	156.0	78.5–177.5
Weight (kg)	47.0	10.7–78.1
MTX dosage (g)	14.0	1.6–20.0
MTX dosage (g/m^2^)	8.0	2.9–14.3
MTX plasma concentration (μmol/L)^a^	51.00	1.02–692.32
White blood cells (/mm^3^)	6,840	90–14,690
Hemoglobin (g/L)	8.6	6.3–12.6
Platelet count (104/mm^3^)	16.4	3.3–39.7
Blood urea nitrogen (mg/dl)	11.1	7.4–57.0
Serum creatinine (mg/dl)	0.81	0.23–3.47
Albumin (g/dl)	3.5	2.3–4.2
Aspartate aminotransferase (U/L)	53.0	17.0–593.0
Alanine aminotransferase (U/L)	72.0	22.0–685.0
Total bilirubin (mg/dl)	0.60	0.30–1.80
Urine pH	7.8	7.0–8.5

a Methotrexate plasma concentrations in the decision for CPG2 treatment.

**Figure 3:**
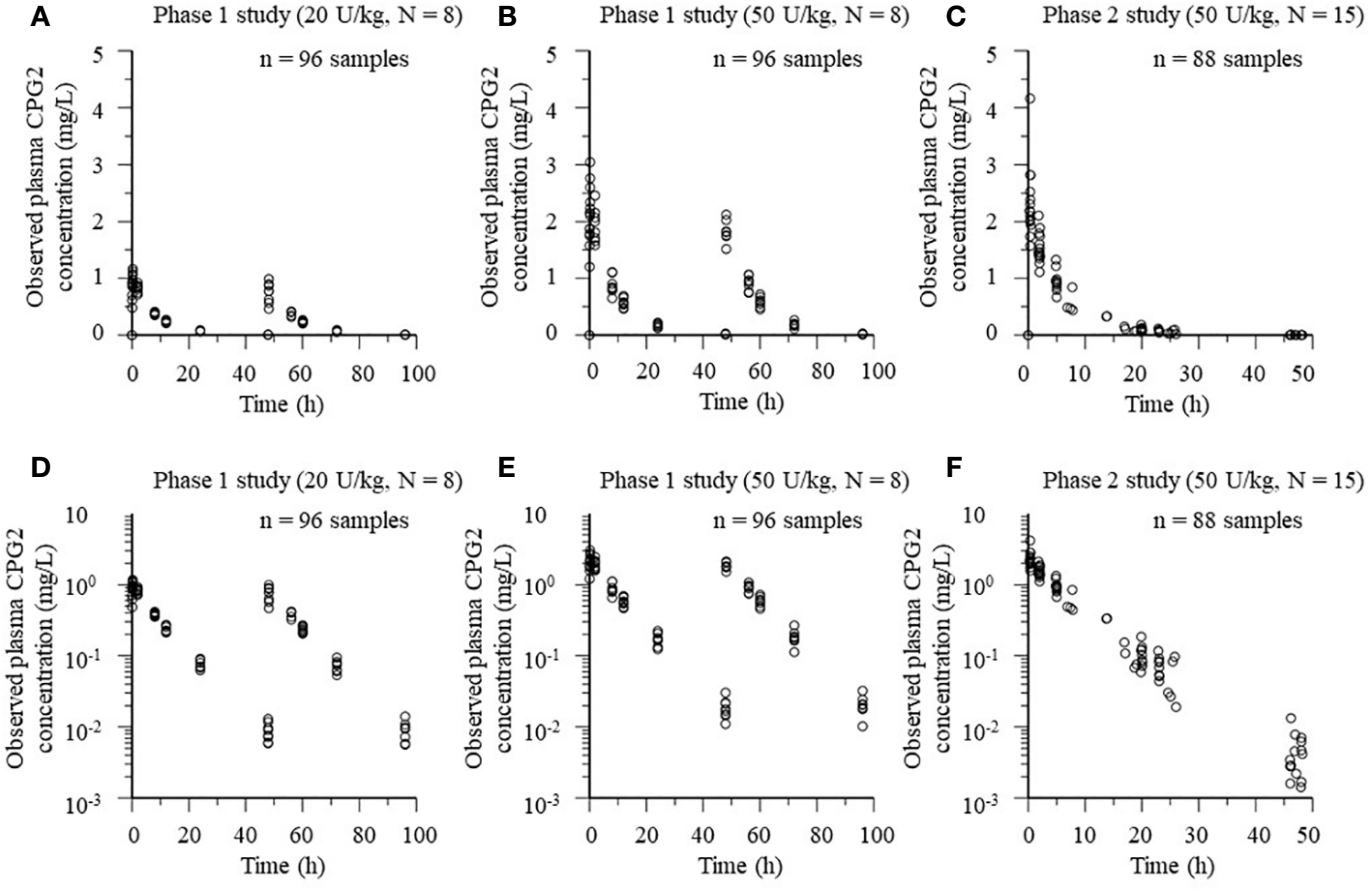
Plots of plasma CPG2 concentrations versus time profile in healthy volunteers (phase 1 study) and patients (phase 2 study). **(A, B, D, E)** Phase 1 study. **(C, F)** Phase 2 study. **(A–C)** Observed plasma CPG2 concentrations versus time. **(D–F)** Observed plasma CPG2 concentrations (logarithmic scale) versus time.

The popPK analysis dataset of CPG2 included 192 plasma samples from 16 healthy volunteers in the phase 1 study. Eighty-eight plasma CPG2 samples and 115 plasma MTX samples were used for the CPG2-MTX popPK-PD modeling, which were obtained from 15 patients in phase 2. Scatter plots of plasma CPG2 concentration versus time profiles in the phase 1 and 2 studies are shown in **Figure 3**.

### 3.2 Development of the popPK and popPK-PD models

#### 3.2.1 PopPK analysis of CPG2 in the phase 1 and 2 studies

A one-compartment model with intravenous infusion of CPG2 was chosen based on the selection analysis of compartmental models in the phase 1 study (**Table 3**). In the covariate modeling process with univariate testing, BSA, body weight, and height as covariates on *CL* and *V* significantly improved the fit of the model in the phase 1 and 2 studies. In the final model, BSA was identified as a statistically significant covariate of *CL* and *V* of CPG2 in the phase 1 and 2 studies. All the popPK parameter estimates for the final model are listed in **Table 4**. The *post-hoc* analysis by the final model showed the mean popPK parameters of CPG2 as follows: *CL* = 0.329 L/h (range, 0.302–0.356) and *V* = 3.17 L (range, 2.97–3.36) in the phase 1 study and *CL* = 0.380 L/h (range, 0.289–0.470) and *V* = 2.04 L (range, 1.47–2.60) in the phase 2 study.

**Table 3:** Covariate screening of CPG2 in the phase 1 and 2 studies.

	Phase 1		Phase 2	
Compartment model	Δ*OFV*	*p*-value	Δ*OFV*	*p*-value
1—compartment (basic model)	NC	NC	NC	NC
2—compartment (basic model)	2.0 × 10^−5^	1	NA	NA
Covariate model	Δ*OFV*	*p*-value	Δ*OFV*	*p*-value
*CL_CPG2_* =*tvCL_CPG2_* x *BSA^θ^*^1^	10.736	0.001	32.826	<0.001
*CL_CPG2_* =*tvCL_CPG2_* x *Body Weight*^θ 1^	9.043	0.003	29.660	<0.001
*CL_CPG2_* =*tvCL_CPG2_* x *Height^θ^*^1^	5.780	0.016	30.225	<0.001
*V_CPG2_* =*tvV_CPG2_* x *BSA^θ^*^1^	10.747	0.001	32.133	<0.001
*V_CPG2_* =*tvV_CPG2_* x *Body Weight^θ^*^1^	9.597	0.002	30.560	<0.001
*V_CPG2_* =*tvV_CPG2_* x *Height^θ^*^1^	4.610	0.032	25.668	<0.001

CPG2, glucarpidase; MTX, methotrexate; BSA, body surface area; tvCL, typical value of clearance; tvV, typical value of distribution volume; NA, not applicable; NC, not calculated; ΔOFV, change of objective function value.

**Table 4:** The final model and bootstrap validation.

CPG2 popPK final model in phase 1	Parameters	Original estimate	Data (95% CI)	Bootstrap median	Estimates (95% CI)
*CL_CPG2_* (L/h)=*tvCL_CPG2_* x *BSA^θ^*^1^ (CV: 3.2%)	*tvCL_CPG2_* (L/h)	0.0590	(0.0244–0.0936)	0.0562	(0.0225–0.1160)
	*θ*1	3.227	(2.153–4.302)	3.178	(1.759–4.970)
*V_CPG2_* (L)=*tvV_CPG2_* x *BSA^θ^*^2^ (CV: 6.4%)	*tvV_CPG2_* (L)	0.957	(0.447–1.467)	0.863	(0.351–1.617)
	*θ*2	2.251	(1.230–3.273)	2.277	(1.148–4.040)
Residual variability (mg/L)		0.170			
CPG2 popPK final model in phase 2	Parameters	Original estimate	Data (95% CI)	Bootstrap median	Estimates (95% CI)
*CL_CPG2_* (L/h)=*tvCL_CPG2_* x *BSA^θ^*^1^ (CV: 17.4%)	*tvCL_CPG2_* (L/h)	0.238	(0.203–0.270)	0.241	(0.209–0.281)
	*θ*1	1.440	(1.105–1.770)	1.425	(1.068–1.733)
*Vd_CPG2_* (L)=*tvV_CPG2_* x *BSA^θ^*^2^ (CV: 22.1%)	*tvV_CPG2_* (L)	1.200	(1.066–1.330)	1.201	(1.010–1.332)
	*θ*2	1.561	(1.391–1.730)	1.557	(1.355–1.812)
Residual variability (mg/L)		0.100			
PopPK-PD final model in phase 2	Parameters	Original estimate	Data (95% CI)	Bootstrap median	Estimates (95% CI)
*CLr_MTX_* (L/h)=*tvCLr_MTX_* x *Body Weight*/*Scr*/60 (CV: 33.5%)	*tvCLr_MTX_* (L/h)	3.248	(1.783–4.712)	2.912	(2.271–4.209)
*Vc_MTX_* (L)=*tvVc_MTX_* x *Body Weight*/*BSA* (CV: 29.1%)	*tvVc_MTX_* (L)	0.386	(0.229–0.543)	0.347	(0.256–0.539)
*Vp_MTX_* (L)=*tvVp_MTX_* x *Body Weight*/60 (CV: 90.6%)	*tvVp_MTX_* (L)	3.052	(1.698–4.405)	3.348	(2.053–5.344)
*Vmax=tva* x [*CPG2 concentration*] (CV: 79.8%)	*tvα* (L/h)	6.545 × 10^5^	(3.666–9.434)	6.242 × 10^5^	(4.276 × 10^5^–8.686 × 10^5^)
*Q* (L/h)		0.0778 (Fixed)			
*Km* (μmol)		86 (Fixed)			
Residual variability		1.414			

CPG2, glucarpidase; MTX, methotrexate; CI, confidence interval; CV, coefficient of variation; BSA, body surface area; Scr, serum creatinine; V_max_, maximum metabolic velocity; tvCLr, typical value of renal clearance; tvCL, typical value of clearance; tvV, typical value of distribution volume; tvVc, typical value of distribution volume in the central compartment; tvVp, typical value of distribution volume in the peripheral compartment; α, conversion constant between V_max_ and catalyst concentration; Km, Michaelis–Menten constant; Q, intercompartmental clearance; θ, covariate as an allometric scale.

#### 3.2.2 PopPK-PD analysis of MTX treated with CPG2 in the phase 2 study

In basic model building, *Q* without interindividual variability made the 95% CI of all other parameters optimal. The estimated *Q* = 0.0632 L/h increased OFV compared with *Q* = 0.0778 L/h, which is the Japanese MTX popPK value reported by Fukuhara et al. Therefore, *Q* = 0.0778 was selected as the fixed value in the exploratory covariate analyses. PK parameters scaled by various subject sizes were used to evaluate the reduction in OFV in the final model. Body weight/serum creatinine ratio was identified as a covariate for both *CLr_MTX_* and *Vp_MTX_*, whereas body weight/BSA ratio was a covariate for *Vc_MTX_*. The final model, including covariates, is presented as follows:


$$\begin{array}{l} CLr_{MTX}\;\left(\text{L}/\text{h}\right)=3.248\times \\ Body\;Weight/Serum\;creatinine/60(\text{CV }33.5\%) \end{array}$$

$$Vc_{MTX}\;\left(\text{L}\right)=0.386\times Body\;Weight/BSA(\text{CV }29.1\%)$$

$$Vp_{MTX}\;\left(\text{L}\right)=3.052\times Body\;Weight/60(\text{CV }90.6\%)$$

$$\alpha =6.545\times 10^{5}(\text{CV }79.8\%)$$

where *α* is a constant to compose a part of the following differential equation:


$$\begin{array}{l} dXc/dt=-\left(Kr+K12\right)\times Xc+K21\times Xp-\\ \alpha \times \left[plasma\;CPG2\;concentration\right]\times \\ \left[plasma\;MTX\;concentration\right]/\\ \left(86\;\text{μmol}/\text{L}+\left[plasma\;MTX\;concentration\right]\right) \end{array}$$

Body weight was standardized at 60 kg (58.4 kg; a representative value of body weight in Japan). The *post-hoc* analysis by the final model showed the mean popPK-PD parameters as follows: *CLr_MTX_* = 2.424 L/h (95% CI: 1.755–3.093), *Vc_MTX_* = 12.6 L (95% CI: 10.8–14.3), *Vp_MTX_* = 2.15 L (95% CI: 1.60–2.70), and *α* = 8.131 x 10^5^ (4.864 x 10^5^–11.398 x 10^5^).

### 3.3 Model validation

**Figure 4:**
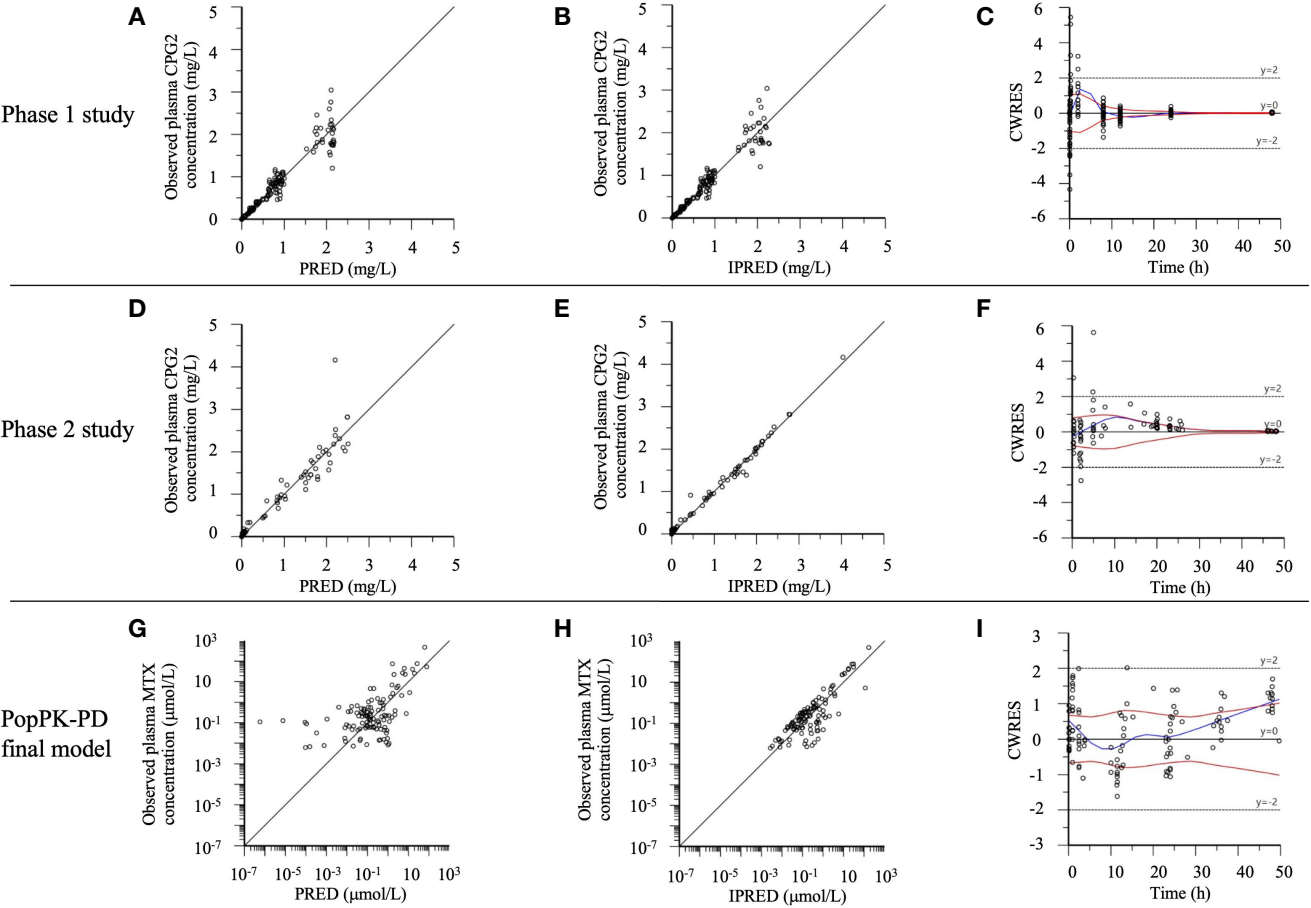
The phase 1 and 2 study CPG2, CPG2-MTX popPK-PD final model goodness-of-fit plots. **(A–C)** Phase 1 study CPG2 final model. **(D–F)** Phase 2 study CPG2 final model. **(G–I)** CPG2-MTX popPK-PD final model. **(A, D)** Observed plasma CPG2 concentrations versus PRED (predicted plasma CPG2 concentrations). **(B, E)** Observed plasma CPG2 concentrations versus IPRED (individual predicted plasma CPG2 concentration). **(C, F, I)** CWRES (conditional weighted residuals) versus time. **(G)** Observed plasma MTX concentrations (logarithmic scale) versus PRED. **(H)** Observed plasma MTX concentrations (logarithmic scale) versus IPRED.

The goodness-of-fit of the final model for the CPG2 popPK and CPG2-MTX popPK-PD in healthy volunteers and patients was evaluated using PRED, IPRED, and CWRES (**Figure 4**). All distributions in the plot of observed concentrations against PRED or IPRED display bilateral symmetry around the regression line of *y* = *x*, since the difference in low plasma MTX concentrations <0.1 μmol/L is within a clinically acceptable range of error. CPG2 CWRES was out of range (−5 to 5) in the phase 1 and 2 studies and was only 2/192 (0.1%) and 1/88 (1.1%), respectively, and that of MTX was zero.

**Figure 5:**
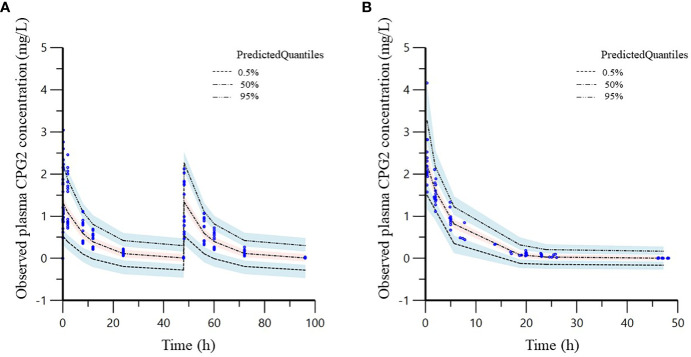
Visual predictive check (VPC) of the phase 1 **(A)** and 2 **(B)** study CPG2 final model. Quantile deviation (blue shaded) obtained from 1,000 datasets using the final model was superimposed on the observed plasma CPG2 concentration of quantile deviation (red shaded). Blue shaded is 95% confidence intervals for the predicted 5th and 95th percentiles. Red shaded is 95% confidence interval for the predicted 50th percentile. The blue cycles show the observed plasma CPG2 concentrations versus time after CPG2 dosing.

VPC plots of CPG2 demonstrated that most observed concentrations were within the 95% prediction intervals of the simulations, and the median lines of the observed and predicted concentrations were similar (**Figure 5**), indicating good predictive performance of the final model.

Furthermore, bootstrap evaluation of the final model almost concluded normally, and the medians of all estimated popPK and popPK-PD parameters were equal to the values of the final model (**Table 4**).

### 3.4 Sampling strategy and performance prediction

**Figure 6:**
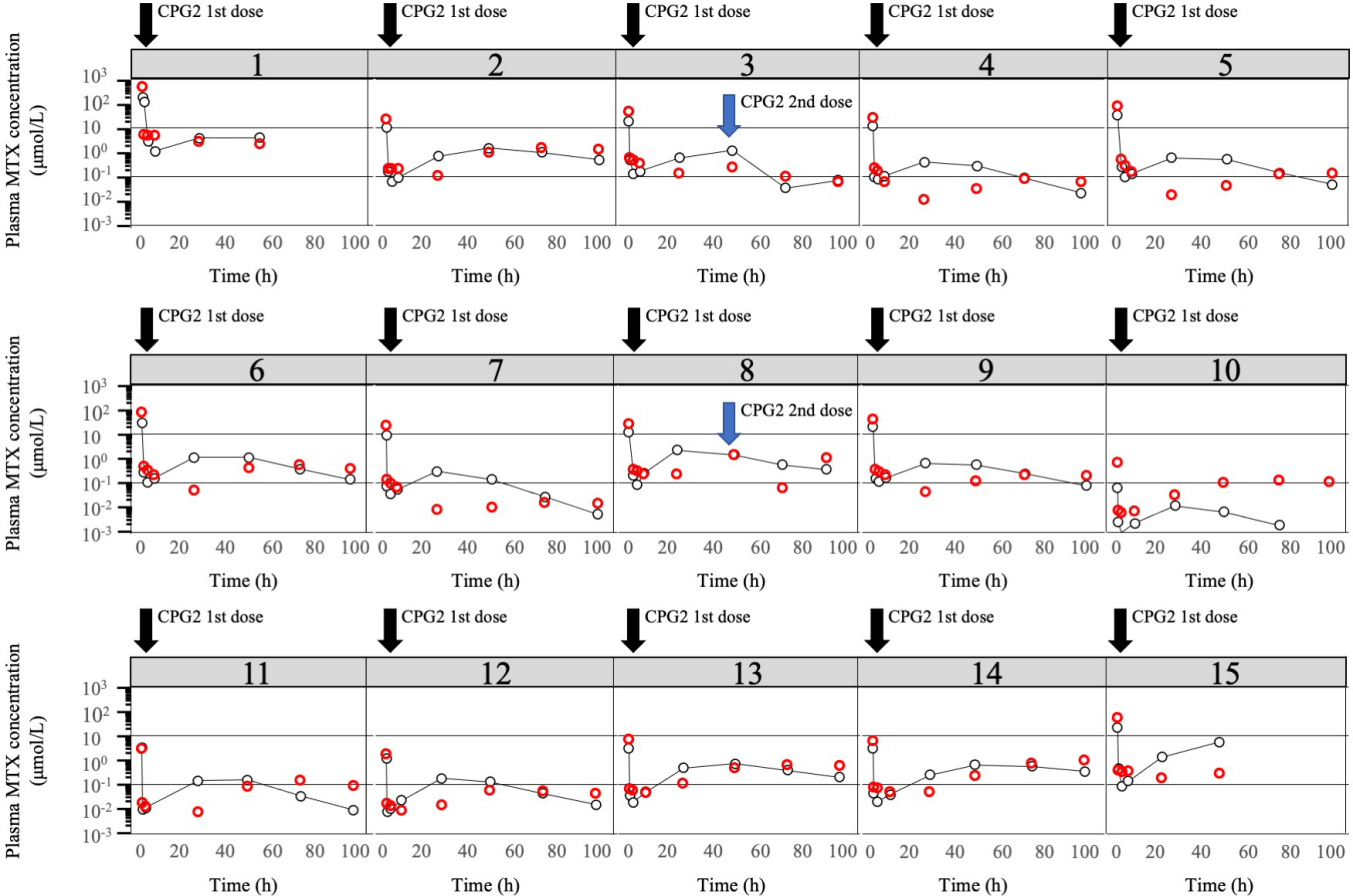
Plot of logarithm of observed plasma MTX concentrations and predicted plasma MTX concentrations. Observed plasma MTX concentrations (red cycle) and predicted plasma MTX concentrations (black cycle) using the CPG2-MTX popPK-PD final model versus time in the phase 2 study individual patients (*N* = 15). The two solid black lines represent plasma methotrexate concentrations of 0.1 and 1 μmol/L, respectively.

**Figure 7:**
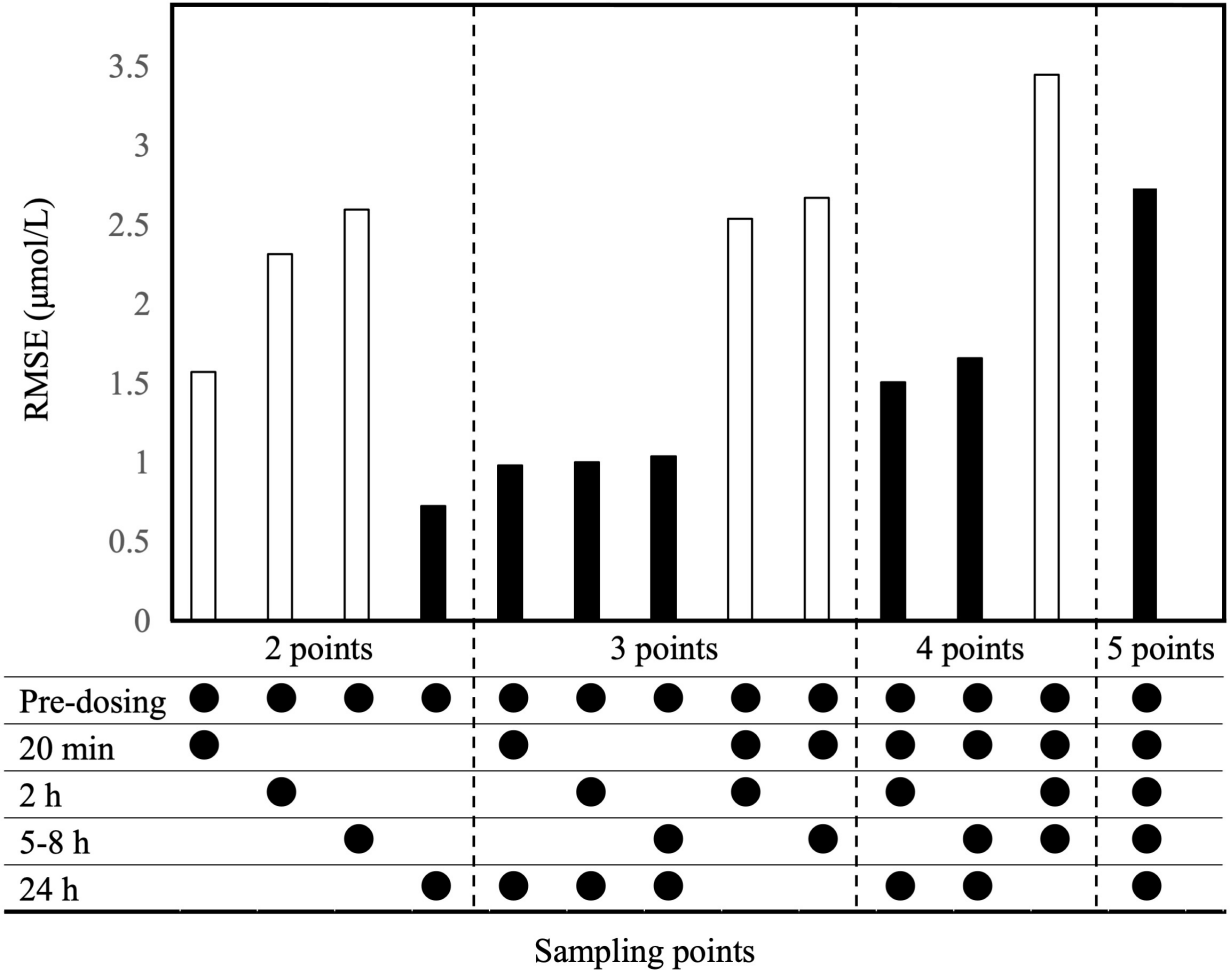
Evaluation of RMSE based on the Bayesian estimation of CPG2-MTX popPK-PD final model. Bayesian estimation was conducted for all combinations of sample points. Sampling points are CPG2 pre-dosing, after dosing at 20 min, and 2, 5–8, and 24 h. White bars exclude 24-h values in the sample points, while black bars include 24-h values.

The observed plasma concentrations of MTX and Bayes-estimated plasma concentrations, calculated using the CPG2-MTX popPK-PD final model versus time for each patient, are presented in **Figure 6**. The plasma MTX concentration versus time curves showed a graphically good reflection of the alterations of reduced and rebound in plasma MTX concentrations after the administration of CPG2 (**Figure 7**). The sampling strategies, including the smallest value of predictive performance, were as follows: 2-point sampling (before CPG2 dosing plus 24 h after dosing) and 3-point sampling (before CPG2 dosing plus 20 min and 24 h after dosing), with ME = −0.056 and 0.03, MAE = 0.458 and 0.543, and RMSE = 0.729 and 0.896, respectively. The relationship between sampling points and RMSE is shown in **Figure 7**.

Additionally, the number of patients with plasma MTX concentrations over and under 1.0 μmol/L was 2 and 13, respectively, at 48 h after the first CPG2 dose. The hit ratio of Bayesian estimation for plasma MTX concentration over or under 1.0 μmol/L was 93.3% (14/15) using the plasma MTX concentrations at pre-CPG2 dosing and 24 h after CPG2 dosing.

## 4 Discussion

This study is the first to report popPK for CPG2 and popPK-PD for MTX with CPG2 in healthy volunteers and in patients treated with MTX. The popPK parameters obtained from the phase 1 study were similar to those previously reported in our non-compartmental analysis: *CL* = 0.291–0.347 L/h and *V* = 3.12–3.67 L (15). The covariate of the final model was BSA in both phase 1 and 2 studies. However, there was a discrepancy in the final model values for *CL* in the phase 1 and 2 studies. Since the phase 1 study showed that the BSA in healthy adults showed small variations compared with those in the phase 2 study including children, the final model of the phase 2 study is considered to reflect more developmental changes. The mean PK parameters normalized by individual body weight in the phase 1 and 2 studies were *CL* = 0.0896 ml/min/kg, *Vd* = 51.9 ml/kg and *CL* = 0.0822 ml/min/kg, *Vd* = 56.0 ml/kg, respectively. The PK parameters in patients with normal and decreased renal function, reported by the PK study PR001-CLN-005 (14), were *CL* = 0.0892 ml/min/kg, *Vd* = 58.0 ml/kg and *CL* = 0.0860 ml/min/kg, *Vd* = 67.9 ml/kg. Since it has been reported that CPG2 has no intracellular translocation, its distribution in the body estimated from the volume of distribution is considered intravascular (14). Since CPG2 is a protein–enzyme preparation with substance properties that are rapidly metabolized regardless of organ function, it was speculated that the population mean PK parameters of CPG2 are not easily affected by pathological conditions. Therefore, no dose adjustment for CPG2 is recommended in patients with organ dysfunction. In addition, there was no significant difference in the parameters between the Japanese and foreigners, suggesting no ethnic differences in the PK of this drug.

The mean Japanese PK parameters of MTX previously reported in adults with almost normal renal function are as follows (5): *CLr_MTX_* = 5.39 L/h, *Vc_MTX_* = 25.7 L, *Vp_MTX_* = 2.62 L, and *Q* = 0.0951 L/h (5). In the phase 2 study, MTX-related renal dysfunction was observed in 57.1% (8/14) of the patients. However, the serum creatinine level, which is one of the indicators of renal function, was 1.23 ± 0.89 mg/dl (mean ± standard deviation) immediately before CPG2 administration, increased to 1.87 ± 1.78 mg/dl on day 4 after CPG2 administration, and decreased to baseline on day 11. These results suggest that the popPK parameters of MTX in this study are appropriate when considering the body size and renal function.

Rebound plasma MTX concentrations were observed in all the patients after the first administration of CPG2. Therefore, more than 48 h of continued monitoring of plasma MTX concentrations is recommended (8). We verified the performance of the Bayes-estimated plasma MTX concentrations calculated using the CPG2-MTX popPK-PD final model. The RMSE values were gradually increased in order of including sampling points of 24 h, 20 min, 2 h, and 5–8 h. As the number of sampling points increased the RMSE value, the predictive performance tended to decrease. The predictive performance was arithmetically superior when using the plasma MTX concentrations at pre-CPG2 dosing and 24 h after CPG2 dosing. Bayesian estimation of the plasma MTX concentrations at 48 h based on the obtained popPK-PD model might be useful for MIPD.

This study has several limitations. First, the estimated plasma MTX concentrations at an early time point after CPG2 dosing, based on the CPG2-MTX popPK final model, tended to be underestimated. Therefore, including sampling points within 8 h decreased the performance of Bayesian estimation of plasma MTX concentrations at 48 h. Second, a fixed value was used for the intercompartmental clearance *Q* of MTX. This might decrease the performance of Bayesian estimation of rebound in plasma MTX concentrations. Third, renal and extrarenal excretion could not be accurately distinguished because the data did not include urine MTX concentrations. In general, renal clearance is expressed using a glomerular filtration rate as covariate, while extrarenal clearance is defined as non-glomerular filtration. Accurately, extrarenal clearance and CPG2 clearance of MTX should be shown, but it is difficult to estimate them separately. Non-renal MTX clearance is significantly affected by CPG2 administration because MTX clearance is reduced in patients with delayed MTX excretion. Therefore, the non-renal MTX clearance model was built with CPG2 and unexpected errors (*n*).

In the phase 2 study, a rebound in plasma MTX concentrations was observed in many cases at a fixed dose (50 U/kg) of CPG2. However, the plasma MTX concentrations could be managed because the timing of the second dose of CPG2 was predictable. These CPG2-MTX popPK analysis and Bayesian estimation of rebound in plasma MTX concentrations may support early decision-making for the second dose of CPG2.

## Data availability statement

The original contributions presented in the study are included in the article/supplementary material, further inquiries can be directed to the corresponding author/s.

## Author contributions

HK and TK contributed significantly to the conceptualization and design of the pharmacokinetic study. KY, HH, YY, AO, JH, KK, AK, YK, and HK conducted this trial and collected clinical data. YF, TK, and YH contributed to data analysis and interpretation. YF drafted the manuscript, which was revised by TK and HH. All authors contributed to the article and approved the submitted version.
